# Examination of the Reliability and Responsiveness of a Japanese Version of the Movement Imagery Questionnaire for Children: Toward Application in Children With Developmental Coordination Disorder

**DOI:** 10.1155/oti/1095914

**Published:** 2026-06-02

**Authors:** Akira Nakashima, Miyu Murata, Honoka Makiyama, Kohei Fukuda, Kyosuke Kawaguchi, Toshio Higashi, Ryoichiro Iwanaga

**Affiliations:** ^1^ Japan Society for the Promotion of Science, Tokyo, Japan, jsps.go.jp; ^2^ Graduate School of Biomedical Sciences, Nagasaki University, Nagasaki, Japan, nagasaki-u.ac.jp; ^3^ Center for Child Mental Health Care and Education, Nagasaki University, Nagasaki, Japan, nagasaki-u.ac.jp

## Abstract

**Objective:**

Developmental coordination disorder (DCD) is a neurodevelopmental condition marked by impaired motor coordination despite the absence of an underlying neurological condition affecting movement (e.g., cerebral palsy, muscular dystrophy, and degenerative disorder). Motor imagery training (MIT) has recently gained attention as a rehabilitation approach for children with DCD. Effective implementation of MIT requires accurate assessment of motor imagery (MI) abilities; however, no comprehensive evaluation tool for children exists in Japan. This study is aimed at developing a Japanese version of the Movement Imagery Questionnaire for Children (MIQ‐C) and examining its reliability and responsiveness.

**Methods:**

Thirty‐three children (18 males and 15 females; mean age 10 ± 0.9 years) participated. Internal consistency was assessed using Cronbach′s alpha. Test–retest and interrater reliability were examined using intraclass correlation coefficients. Responsiveness was evaluated by examining changes in MIQ‐C scores before and after training tasks.

**Results:**

The Japanese version of the MIQ‐C demonstrated strong internal consistency (Cronbach′s *α* = 0.82). Test–retest and interrater reliability showed moderate to strong agreement. No significant changes were observed in response to specific tasks, supporting appropriate responsiveness.

**Conclusions:**

The Japanese version of the MIQ‐C is a reliable and valid instrument for assessing MI abilities in Japanese children, providing a useful tool to support the implementation of MIT in clinical and educational settings.

## 1. Introduction

Developmental coordination disorder (DCD) is a neurodevelopmental condition affecting 5%–8% of school‐aged children [1–3]. It is characterized by impairments in balance, coordination, and motor skills, such as handwriting [4, 5], which hinder daily activities [6, 7]. Children with DCD frequently present with co‐occurring mental health conditions [8, 9] and are at increased risk of social withdrawal [10]. DCD is often diagnosed after the age of 5, when motor difficulties become more apparent. Early identification and timely intervention are considered beneficial, as they may support the development of functional abilities and adaptive skills.

Motor imagery training (MIT) has emerged as a promising rehabilitation strategy for children with DCD. MIT involves the repeated use of motor imagery (MI) to enhance task performance. Its efficacy is well established in patients with stroke [11, 12] and has also been reported in children with DCD [13–16]. In our scoping review investigating MIT tasks used for children with DCD, we found that the majority of participants who received MIT were between 7 and 12 years of age. During MIT, MI tended to be performed using kinesthetic imagery, and many studies employed a method in which children observed video clips of the MIT tasks while generating MI. In clinical studies, approximately half did not assess the MI ability of the participating children [17]. To implement MIT effectively, however, it is essential to assess MI abilities accurately [18]. Conventional assessments such as mental chronometry and mental rotation have been employed in children, but these methods are limited in scope and subject to learning effects [19–21]. In addition, these methods do not enable evaluation from MI perspectives (e.g., internal visual imagery and external visual imagery) and, therefore, cannot comprehensively assess overall MI ability. To address these limitations, Martini et al. developed the Movement Imagery Questionnaire for Children (MIQ‐C), adapted from the Motor Imagery Questionnaire‐3 (MIQ‐3) for adults. The MIQ‐C evaluates MI across three dimensions: internal visual imagery, external visual imagery, and kinesthetic imagery [22]. The MIQ‐C has been translated into Persian, Slovenian, Turkish, and Russian [23–26].

Currently, no validated instrument is available in Japan to comprehensively assess MI abilities in children across these three domains. Establishing such a tool would enhance the application of MIT in developmental rehabilitation.

Therefore, this study is aimed at developing a Japanese version of the MIQ‐C and examining its reliability and responsiveness with reference to the COSMIN guidelines for evaluating measurement properties. The completed COSMIN checklist is provided in Table S1.

## 2. Materials and Methods

### 2.1. Participants

Thirty‐three typically developing children were included in the study. The sample size was based on prior research employing G∗Power for similar studies, which indicated that 20 or more participants were sufficient [27]. Participants were aged 9–12 years and had no medical diagnosis of autism spectrum disorder or attention‐deficit/hyperactivity disorder (ADHD). The study was conducted in collaboration with an after‐school child care center in Nagasaki. Written explanations of the study′s purpose were provided to the participants and their parents, and informed consent was obtained. This study was approved by the Ethics Committee of the Graduate School of Biomedical Sciences, Nagasaki University (No. 24111405; approved on November 14, 2024). This study protocol was not prospectively registered, as it was an observational study focused on the evaluation of measurement properties.

### 2.2. Translation Procedure

When adapting the MIQ‐C for Japanese children, both linguistic and cultural considerations were taken into account to ensure that the items were understandable and conceptually equivalent within the Japanese context. Two researchers with expertise in movement imagery and proficiency in English first conducted a forward translation from English to Japanese. A third researcher with expertise in developmental disorders then joined the process. Together, the three researchers reviewed the translated version to ensure conceptual accuracy and cultural appropriateness for children. A backward translation was subsequently performed by an English proofreading company (Editage), translating the Japanese version back into English. Rose Martini, the developer of MIQ‐C, reviewed the translation, and the Japanese version of the MIQ‐C was finalized.

### 2.3. Evaluation

#### 2.3.1. Japanese Version of the MIQ‐C

The MIQ‐C, developed by Martini et al., is an adaptation of the MIQ‐3 for children. It is a questionnaire assessing MI ability through three forms of MI—internal visual, external visual, and kinesthetic—for four movements: standing on one foot, shoulder joint horizontal adduction, trunk flexion, and jumping. Responses are rated on a 7‐point Likert scale.

#### 2.3.2. Performance Test

Performance was assessed using the ball rotation task (BRT), a task designed to facilitate early motor learning. Participants were instructed to rotate two golf balls counterclockwise in their palms, with the number of rotations serving as the performance indicator [28]. The BRT has been widely used in previous studies and is recognized as a reliable measure of performance [29–32]. In the present study, participants performed the task while seated comfortably on the floor. The number of rotations completed within 1 min was recorded before and after BRT physical training. During BRT physical training, participants performed the task while observing a demonstration video displayed on a tablet.

### 2.4. Experimental Procedures

**Figure 1:**
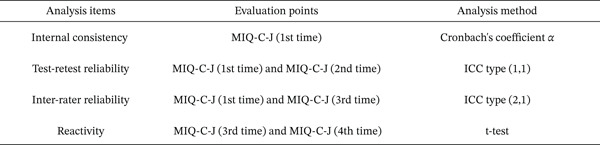
Evaluation and statistical methods applied in each analysis. MIQ‐C‐J: Japanese version of the Movement Imagery Questionnaire for Children; ICC: intraclass correlation coefficient.

The study was conducted across 3 days. The Japanese version of the MIQ‐C was administered once at the first assessment, once at the second, and twice at the third, resulting in four measurements. Each assessment day was separated by 1–3 weeks, which is commonly recommended in psychometric research to minimize recall bias while avoiding substantial changes in the measured construct over time [33]. Data from these assessments were used to evaluate the reliability and responsiveness of the Japanese version of the MIQ‐C (Figure 1). All assessments were conducted individually in a quiet environment with one researcher and one participant. Five occupational therapists served as researchers.

#### 2.4.1. Internal Consistency of the Japanese Version of the MIQ‐C

Internal consistency was examined by calculating Cronbach′s alpha coefficient for the MIQ‐C subscales at the initial evaluation. Internal consistency is considered acceptable at ≥ 0.7, good at ≥ 0.8, and excellent at ≥ 0.9.

#### 2.4.2. Retest Reliability of the Japanese Version of the MIQ‐C

Retest reliability was assessed using intraclass correlation coefficients (ICCs) [1] when the same examiner conducted the initial and second evaluations. The interpretation of ICC values was based on the guidelines proposed by Koo and Li; values < 0.50 were considered poor, 0.50–0.75 moderate, 0.75–0.90 good, and > 0.90 excellent [34].

#### 2.4.3. Interrater Reliability of the Japanese Version of the MIQ‐C

Interrater reliability was tested by having a different examiner conduct the third evaluation, followed by the calculation of ICC [1, 2]. The interpretation of ICC values was based on the guidelines proposed by Koo and Li; values < 0.50 were considered poor, 0.50–0.75 moderate, 0.75–0.90 good, and > 0.90 excellent [34].

#### 2.4.4. Responsiveness of the Japanese Version of the MIQ‐C

**Figure 2:**
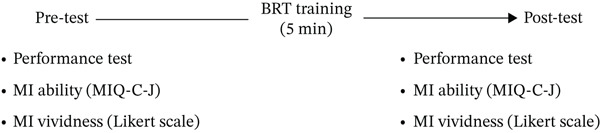
Procedures for assessing reactivity. MI: motor imagery; BRT: ball rotation task; MIQ‐C‐J: Japanese version of the Movement Imagery Questionnaire for Children.

Responsiveness was evaluated at the third assessment based on the hypothesis that “the vividness of MI for specific tasks improves before and after motor learning, whereas no change occurs in overall MI ability as assessed by the Japanese version of the MIQ‐C.” The BRT was used for this evaluation. Participants first completed the MIQ‐C assessment, and then carefully observed a video of the BRT. They performed the BRT for 1 min, followed by 1 min of imagining the task, after which they rated the clarity of their imagery on a Likert‐type rating scale. Next, after 5 min of physical practice of the BRT, the BRT performance test, MI vividness rating, and MIQ‐C assessment were conducted again (Figure 2). Responsiveness was analyzed by comparing MIQ‐C scores, BRT performance, and MI vividness ratings before and after practice using a paired *t*‐test. Exploratory analyses were also conducted to examine the relationship between MIQ‐C subscale scores and task‐specific imagery vividness ratings assessed using a Likert‐type rating scale during the BRT.

### 2.5. Statistical Analysis

Continuous variables were expressed as mean ± standard deviation, and categorical variables are presented as frequencies and percentages. Internal consistency of the Japanese version of the MIQ‐C was assessed using Cronbach′s alpha coefficients for each subscale. Test–retest reliability was evaluated using ICC (Type 1,1), and interrater reliability was assessed using ICC (Type 2,1). The standard error of measurement and minimal detectable change at the 95% confidence level (MDC95) were calculated based on the ICC (1,1) values and standard deviations from the initial assessment. Exploratory convergent validity was examined using Pearson′s correlation coefficients between MIQ‐C subscale scores and task‐specific imagery vividness ratings assessed using a Likert‐type rating scale. Correlation coefficients were interpreted as weak (*r* < 0.40), moderate (0.40 ≤ *r* < 0.70), or strong (*r* ≥ 0.70). Responsiveness was evaluated by comparing MIQ‐C scores, BRT performance, and MI vividness ratings before and after practice using paired *t*‐tests. All statistical analyses were performed using SPSS Version 22.0 (IBM Corp., Armonk, New York, United States), and the significance level was set at *p* < 0.05.

## 3. Results

### 3.1. Basic Attributes

The study included 33 children (18 males and 15 females) with a mean age of 10.0 ± 0.9 years (range: 9–12 years). All participants completed three assessments at intervals of 1–3 weeks. Table 1 presents the MIQ‐C Japanese version scores for each participant at the first assessment.

**Table 1:** Mean and standard deviation of each MIQ‐C Japanese version subscale at each assessment point.

	1st time	2nd time	3rd time	4th time
Internal visual imagery	5.1 ± 0.8	5.4 ± 1.1	5.3 ± 1.5	6.3 ± 6.2
External visual imagery	5.3 ± 1.0	5.5 ± 1.0	5.4 ± 1.5	5.5 ± 1.4
Kinesthetic imagery	4.3 ± 1.3	4.8 ± 1.1	4.8 ± 1.4	5 ± 1.5

### 3.2. Internal Consistency of the Japanese Version of the MIQ‐C

Cronbach′s alpha coefficients were 0.48 for internal visual imagery, 0.81 for external visual imagery, 0.86 for kinesthetic imagery, and 0.82 for all items. These results indicated acceptable to good internal consistency, although the internal visual imagery subscale showed lower internal consistency.

### 3.3. Reliability of the Japanese Version of the MIQ‐C (Test–Retest Reliability)

The ICCs (1,1) were 0.45 (95% CI [0.14, 0.68]) for internal visual imagery, 0.70 (95% CI [0.48, 0.84]) for external visual imagery, and 0.54 (95% CI [0.24, 0.73]) for kinesthetic imagery, indicating moderate test–retest reliability. The SEM and MDC95 values were 0.64 and 1.77 for internal visual imagery, 0.59 and 1.64 for external visual imagery, and 0.91 and 2.53 for kinesthetic imagery, respectively.

### 3.4. Interrater Reliability of the Japanese Version of the MIQ‐C

The ICCs [1, 2] were 0.45 (95% CI [0.13, 0.68]) for internal visual imagery, 0.76 (95% CI [0.56, 0.87]) for external visual imagery, and 0.69 (95% CI [0.45, 0.83]) for kinesthetic imagery, indicating moderate to good interrater reliability.

### 3.5. Exploratory Convergent Validity of the Japanese Version of the MIQ‐C

Exploratory correlation analyses between MIQ‐C subscale scores and task‐specific imagery vividness ratings assessed using a Likert‐type rating scale revealed a significant weak correlation only for the kinesthetic imagery subscale (*r* = 0.354, *p* = 0.047). No significant correlations were observed for the internal visual imagery subscale (*r* = 0.258, *p* = 0.206) or external visual imagery subscale (*r* = 0.233, *p* = 0.217).

### 3.6. Responsiveness of the Japanese Version of the MIQ‐C

Responsiveness was assessed by comparing MIQ‐C scores, BRT performance, and MI vividness ratings before and after BRT practice, as well as the clarity of MI during BRT using a Likert‐type rating scale. No significant differences were observed in MI ability assessed using the Japanese version of the MIQ‐C before and after motor practice, including internal visual imagery (*t*(32) = −0.97, *p* = 0.330), external visual imagery (*t*(32) = −1.83, *p* = 0.070), and kinesthetic imagery (*t*(32) = −1.63, *p* = 0.110). In contrast, BRT performance (*t*(32) = −7.28, *p* < 0.001, Cohen′s dz = 1.27) and imagery vividness ratings (*t*(32) = −4.82, *p* < 0.001, Cohen′s dz = 0.84) showed significant improvements after practice. These findings support the responsiveness of BRT performance and imagery vividness, although changes in MIQ‐C scores were not statistically significant.

## 4. Discussion

This study is aimed at verifying the reliability and responsiveness of the Japanese version of the MIQ‐C, an instrument for assessing MI ability in children. Findings demonstrated sufficient internal consistency, moderate to strong test–retest and interrater reliability, and appropriate responsiveness. These results indicate that the Japanese version of the MIQ‐C is an appropriate tool for evaluating MI ability in children.

The present findings also have important practical implications. The Japanese version of the MIQ‐C provides a useful tool for assessing MI ability in Japanese children in research and clinical settings. Because MI plays an important role in motor learning and rehabilitation, the availability of a culturally adapted questionnaire may facilitate the evaluation of imagery ability in children. Furthermore, this instrument may support future studies investigating MI characteristics in children with neurodevelopmental conditions, such as DCD, and may contribute to the development of imagery‐based rehabilitation and training programs.

Cronbach′s alpha coefficients in the present study were 0.48 for internal visual imagery, 0.81 for external visual imagery, and 0.86 for kinesthetic imagery. Previous studies have reported Cronbach′s alpha values of approximately 0.90 for each subscale, and in the present study, a low coefficient was observed only for internal visual imagery [24]. Validation studies of the MIQ‐C in other languages have generally reported high internal consistency across all subscales. Therefore, the lower Cronbach′s alpha observed for internal visual imagery in the Japanese version may represent a characteristic specific to the present sample or context. The lower Cronbach′s alpha for the internal visual imagery subscale may be attributable to the inherently higher cognitive and sensorimotor demands of generating internal visual imagery. Previous neuroimaging studies have shown that internal visual imagery requires greater activation of sensorimotor regions compared with external visual imagery, indicating that the generation of internal imagery imposes a higher processing load [35]. Such increased demands may reduce the consistency with which children are able to generate vivid internal images across items, thereby lowering interitem correlations and resulting in a reduced alpha coefficient. In addition, MI ability in children shows considerable developmental variability, which may have contributed to inconsistent responses specifically within this subscale [21]. Although cultural or educational factors are unlikely to explain the lower coefficient observed in Japanese children, further research is needed to clarify contributing factors such as item difficulty, age‐related differences, and the developmental trajectory of internal visual imagery ability. However, because the Cronbach′s alpha coefficient for the total scale was high and the coefficients for external visual imagery and kinesthetic imagery were also high, this issue is unlikely to substantially limit the practical use of the Japanese version of the MIQ‐C. [25, 26].

Test–retest reliability results showed poor to moderate reliability for internal visual imagery (0.45), external visual imagery (0.70), and kinesthetic imagery (0.50). In the original MIQ‐C, ICC values were 0.72 for internal visual imagery, 0.43 for external visual imagery, and 0.82 for kinesthetic imagery, indicating relatively lower reliability for external visual imagery [21]. The Turkish version reported higher ICCs (0.94, 0.93, and 0.96, respectively) [14], whereas the Slovenian version also demonstrated high reliability (0.92, 0.90, and 0.90) [23]. Although the Japanese version showed somewhat lower ICC values than the Turkish and Slovenian versions, the results remain sufficient for reliably assessing MI ability in children.

Interrater reliability in the present study ranged from poor to good interrater reliability, with ICC values of 0.45 for internal visual imagery, 0.75 for external visual imagery, and 0.68 for kinesthetic imagery. Previous validation studies of other language versions of the MIQ‐C have not assessed interrater reliability. However, we considered that the manner in which examiners explain how to perform MI and how the imagery should appear could influence children′s responses when administering the MIQ‐C. Therefore, we evaluated interrater reliability in this study. The results indicated that the Japanese version of the MIQ‐C has adequate interrater reliability.

In the responsiveness analysis, we hypothesized that “the vividness of MI for tasks improves before and after BRT motor learning, whereas no changes occur in MI ability as assessed by the Japanese version of the MIQ‐C.” The findings supported this hypothesis: BRT performance and MI vividness improved after training, but MIQ‐C scores did not change. In this study, MI ability was assessed using the Japanese version of MIQ‐C, and vividness was rated using a Likert‐type rating scale. The results suggest that improvements in task performance and experiential learning during BRT practice primarily enhance vividness rather than fundamental MI ability. Similar findings have been reported in studies of athletes, where MI training improved task‐specific vividness [36–38], whereas scores on the MIQ remained unchanged [39]. MI ability represents a basic cognitive capacity, which may not be altered by short‐term task‐specific training. These results indicate that the Japanese version of the MIQ‐C demonstrates appropriate responsiveness and is a reliable assessment tool.

In the exploratory convergent validity analysis, only the kinesthetic imagery subscale showed a significant correlation with task‐specific imagery vividness ratings assessed using a Likert‐type rating scale. However, the MIQ‐C and the task‐specific imagery vividness ratings used in this study were designed to assess different aspects of MI. Specifically, the MIQ‐C evaluates general MI ability across multiple imagery domains, whereas the task‐specific imagery vividness ratings assess task‐specific imagery vividness during the BRT task. Therefore, the limited correlations observed in this study may reflect that the two measures assess partially distinct constructs of MI.

This study has some limitations. First, only typically developing children were included in this study; children with DCD or ADHD, who may benefit from MIT, were not examined, as this study was designed as an initial step to evaluate the measurement properties of the Japanese version of the MIQ‐C in a nonclinical population. Second, the relatively small sample size limited the scope of statistical analyses, including exploratory factor analysis or confirmatory factor analysis. Although an exploratory convergent validity analysis was conducted using task‐specific imagery vividness ratings assessed with a Likert‐type rating scale, the MIQ‐C and the task‐specific imagery vividness ratings assess related but not identical aspects of MI. Therefore, further studies using multiple standardized MI assessments and larger samples are needed to more comprehensively examine the construct validity of the Japanese version of the MIQ‐C. Future research should also examine the applicability of the Japanese version of the MIQ‐C across different age groups and clinical populations, as well as investigate its usefulness in evaluating changes in MI ability following motor learning or rehabilitation interventions. In addition, the present study assessed only short‐term responsiveness; therefore, future studies are needed to examine long‐term responsiveness and the sensitivity of the instrument over extended periods.

## 5. Conclusion

This study examined the reliability and responsiveness of the Japanese version of the MIQ‐C. The results confirmed sufficient reliability and responsiveness, indicating that the instrument is appropriate for evaluating MI ability in Japanese children. The findings are expected to inform future research on MI in children and contribute to progress in sports science and rehabilitation.

## Funding

This study was funded by the Japan Society for the Promotion of Science, 10.13039/501100001691, 24KJ1821.

## Conflicts of Interest

The authors declare no conflicts of interest.

## Supporting information


**Supporting Information** Additional supporting information can be found online in the Supporting Information section. Table S1: COSMIN checklist used to evaluate the measurement properties assessed in this study.

## Data Availability

The data that support the findings of this study are available from the corresponding author upon reasonable request.
